# Noninvasive biomarkers for the detection of GERD-induced pulmonary injury

**DOI:** 10.1007/s00464-024-11180-4

**Published:** 2024-09-12

**Authors:** Andrés R. Latorre-Rodríguez, Sumeet K. Mittal, Ranjithkumar Ravichandran, Austin Reynolds, Andrés Isaza-Restrepo, Jahanvi Mittal, Mary F. Hahn, Ross M. Bremner, Thalachallour Mohanakumar

**Affiliations:** 1 Norton Thoracic Institute, St. Joseph’s Hospital and Medical Center, 500 W Thomas Road, Phoenix, AZ 85013 USA; 2 Grupo de Investigación Clínica, Escuela de Medicina y Ciencias de La Salud, Universidad del Rosario, Bogotá, D.C Colombia; 3 Creighton University School of Medicine, Phoenix Health Sciences Campus, Phoenix, AZ USA; 4 Department of Pathology, St. Joseph’s Hospital and Medical Center, Phoenix, AZ USA

**Keywords:** Gastroesophageal reflux, Respiratory aspiration, Lung injury, Extracellular vesicles, Proof of concept study, Biomarkers

## Abstract

**Background:**

The role of gastroesophageal reflux in progressive lung damage is increasingly recognized. We have proposed, based on our work with lung transplant recipients, a novel immune mechanism of pulmonary injury after aspiration of gastric contents, during which higher levels of normally sequestered lung self-antigens (SAgs) collagen V (Col-V) and K-alpha-1 tubulin (Kα1T) in circulating small extracellular vesicles (EVs) induce the production of self-antibodies (SAbs) anti-Col-V and anti-Kα1T. Thus, we aimed to determine whether levels of SAbs or SAgs increased in an animal model of aspiration-induced lung damage in a nontransplant setting.

**Methods:**

We created a murine model of repetitive lung aspiration using C57BL/6J mice. Mice were aspirated weekly with 1 mL/kg of hydrochloric acid (n = 9), human gastric contents (n = 9), or combined (1:1) fluid (n = 9) once, three, or six times (n = 3 in each subgroup; control group, n = 9). Blood samples were periodically obtained, and all animals were sacrificed at day 90 for pathological assessment. SAbs were measured using an enzyme-linked immunosorbent assay; SAgs and NF-κB contained in small EVs were assessed by western blot.

**Results:**

Aspirated mice weighed significantly less than controls throughout the study and had histological evidence of pulmonary injury at day 90. Overall, aspirated mice developed higher concentrations of anti-Col-V at day 28 (53.9 ± 28.7 vs. 29.9 ± 4.5 ng/mL, p < 0.01), day 35 (42.6 ± 19.8 vs. 28.6 ± 7.2 ng/mL, p = 0.038), and day 90 (59.7 ± 27.7 vs. 34.1 ± 3.2 ng/mL, p = 0.014) than the control group. Circulating small EVs isolated from aspirated mice on day 90 contained higher levels of Col-V (0.7 ± 0.56 vs. 0.18 ± 0.6 m.o.d., p = 0.009) and NF-κB (0.42 ± 0.27 vs. 0.27 ± 0.09 m.o.d., p = 0.095) than those from controls.

**Conclusions:**

This experimental study supports the theory that gastroesophageal reflux leads to the development of lung damage and an increase of humoral markers that may serve as noninvasive biomarkers to detect asymptomatic lung injury among patients with gastroesophageal reflux disease.

**Supplementary Information:**

The online version contains supplementary material available at 10.1007/s00464-024-11180-4.

Gastroesophageal reflux disease (GERD) is a chronic debilitating condition reaching epidemic proportions in the Western world [1]. It is widely accepted that the retrograde flow of gastric contents through an incompetent esophagogastric junction can reach the oropharynx and subsequently be aspirated into the lungs. Moreover, it is increasingly recognized that a broad spectrum of chronic pulmonary diseases (e.g., chronic cough, recurrent bronchitis, aspiration pneumonia, interstitial pulmonary fibrosis, and asthma) are associated with GERD [2, 3]. However, due to the lack of reliable, noninvasive, and standardized methods to detect early lung damage, aspiration-induced pulmonary injury is clinically unsuspected until the advanced stages of pulmonary disease [4].

The implications of aspiration-related lung injury have become even more serious over the last three decades given the increased use of acid suppression medications. These medications (proton pump inhibitors, histamine H2-receptor antagonists, etc.) reduce the acidity of refluxed material, thereby eliminating the most common clinical symptom of GERD (i.e., heartburn), leading people to believe that the disease is cured even though there is continued regurgitation and potential aspiration of gastric contents [5].

The pathophysiology behind GERD-related lung injury has been explored using in vivo models but remains largely unknown. Most studies have reported that acute aspiration leads to worse perfusion, inflammatory cellular infiltration of lung parenchyma, and higher levels of proinflammatory cytokines and growth factors (e.g., TGF-β) related to fibrosis [6–9]. Moreover, after repetitive aspiration in animal models, chronic inflammation develops, characterized by the presence of macrophages and CD4 + and CD8 + cells as well as increased levels of TGF-β, TNF-α, and other proinflammatory cytokines (e.g., IL-1α, IL-1β, IL-2) [10, 11].

Our group has described a novel pathophysiological model proposing an immune mechanism of GERD-induced lung damage among patients with end-stage respiratory disease (i.e., lung transplant candidates and recipients) [12]. Briefly, patients with GERD are at risk of silent, repetitive micro-aspiration of gastric contents. Once the refluxate reaches the lower airway, it is deposited in the alveoli and disrupts the respiratory epithelium, exposing cryptic normally sequestered lung self-antigens (SAgs) such as collagen-V (Col-V) and K-alpha-1 tubulin (Kα1T). Once the SAgs are exposed, the immune system identifies and targets them, resulting in a direct or indirect immune cascade, ultimately leading to the generation of self-antibodies (SAbs) against SAgs that can potentiate the mechanical damage that has already been caused by the gastric contents [13]. Over time, this process leads to chronic inflammation, fibrosis, and undesirable outcomes in lung transplant recipients such as bronchiolitis obliterans syndrome or chronic lung allograft dysfunction [12, 14–16].

We hypothesize that serum concentrations of SAbs and circulating levels of SAgs contained in small extracellular vesicles (EVs) may be early humoral indicators of active though subclinical pulmonary parenchymal damage (i.e., patients with GERD without established lung disease). To validate this proof of concept, we sought to determine whether repetitive micro-aspiration induces lung damage and whether levels of SAbs, SAgs, or proinflammatory markers such as nuclear factor-κB (NF-κB) increase in a murine model of aspiration-related lung damage.

## Methods

### Study design and settings

This preclinical proof of concept study used an experimental animal model to measure circulating levels of SAgs and SAbs (i.e., humoral markers of lung damage) after noninvasive, repetitive aspiration events simulating gastroesophageal reflux. This study was conducted between March 2023 and October 2023 at the Norton Thoracic Institute Research Laboratory and was approved by the Institutional Animal Care and Use Committee (IACUC) of St. Joseph’s Hospital and Medical Center, Phoenix, AZ (Animal Welfare Assurance #A3519-01, Protocol: 628; date of approval: January 25, 2023). The ARRIVE 2.0 guidelines and checklist were followed to ensure a transparent and thorough manuscript (Supplementary Material S1).

### Mice and study groups

Thirty-six, 12-week-old male C57BL/6J mice weighing 20–30 g each were used (The Jackson Laboratory, Bar Harbor, Maine). The C57BL/6J strain was selected due to previous studies confirming the feasibility of inducing pulmonary damage after the administration of SAbs against SAgs in a murine model of orthotopic lung transplantation [17]. All mice were housed in a pathogen-free environment, and all procedures were conducted with sterile precautions approved by the local IACUC. The animals in this study received humane care per the Guide for the Care and Use of Laboratory Animals.

**Fig. 1:**
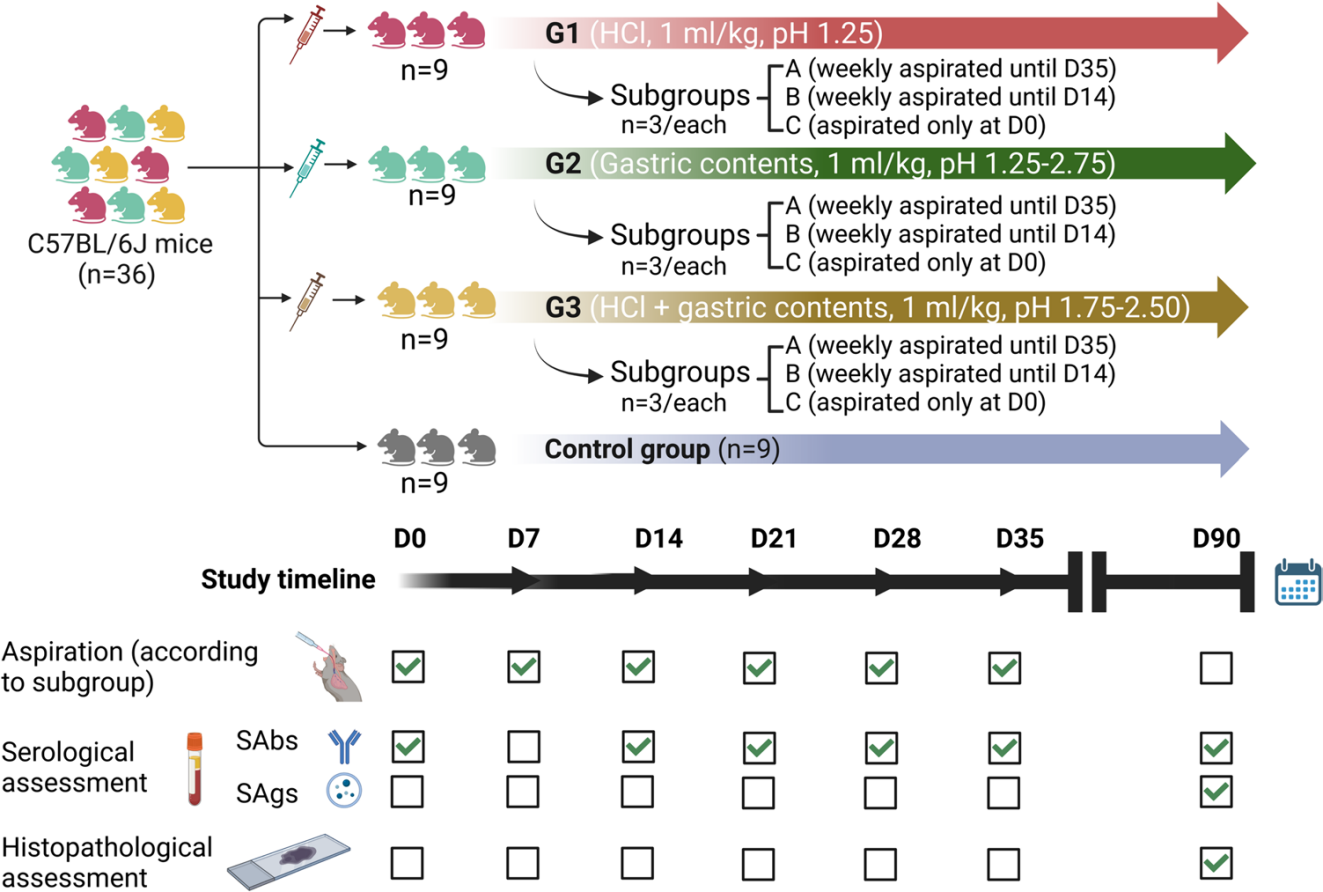
Study flow diagram. Abbreviations: D, day; HCL, hydrochloric acid; SAbs, self-antibodies; SAgs, self-antigens

Four experimental groups (n = 9/each) were defined. Group 1 underwent aspiration with hydrochloric acid (HCl), group 2 with human gastric contents, and group 3 with a 1:1 mixture of human gastric contents and HCl. Group 4 served as a control; importantly, 3 mice within the control group underwent sham aspiration (i.e., instillation of 0.2 mL of air) while the remaining 6 mice did not undergo any treatment. Figure 1 provides a study flow diagram and timeline.

### Aspiration model and substance preparation

#### Aspiration procedure

**Fig. 2:**
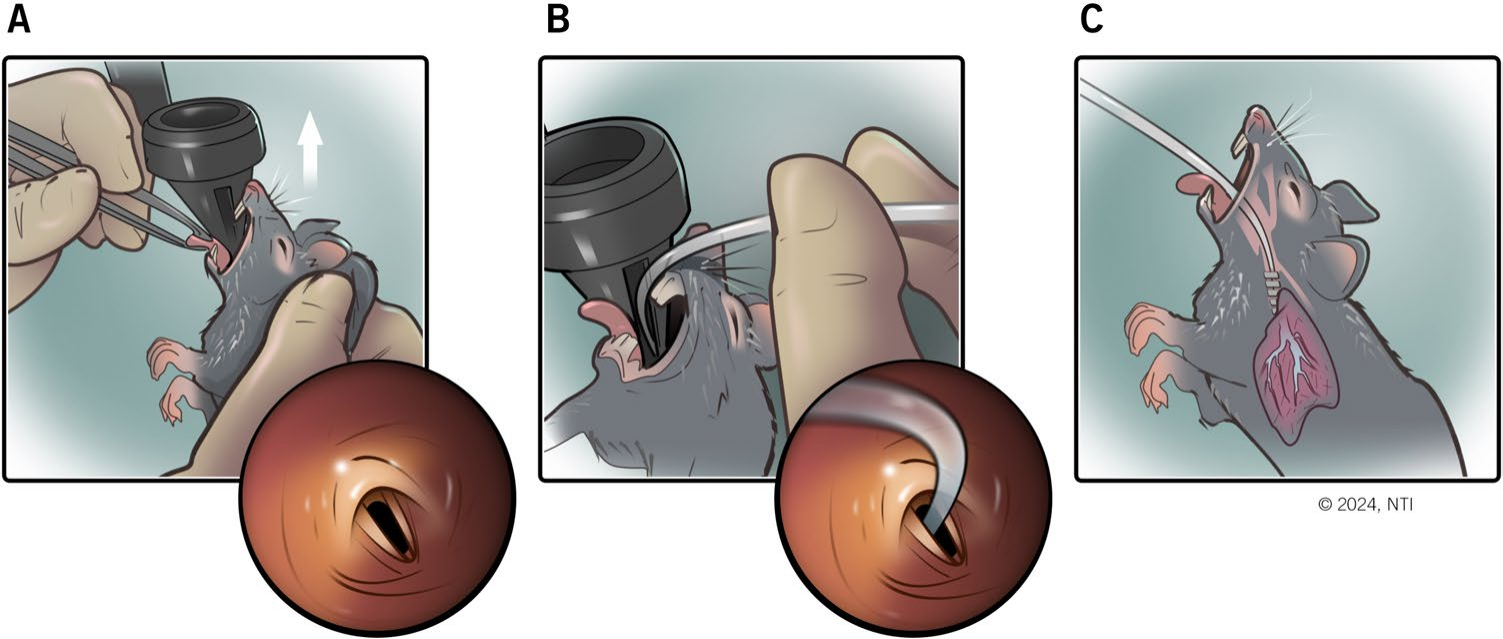
Mice aspiration procedure. **A** The mouse is gently pulled vertically by the scruff onto the speculum of the otoscope, and visualization of vocal cords is confirmed. **B** A 20G sterile plastic catheter (i.e., intratracheal tube) is inserted into the opening on the side of the speculum and advanced beyond the vocal cords. **C** After adequate catheter placement confirmation, the head of the mouse in an upright position was directed upward at a 35–45° angle, and the substance is instilled into the trachea using a 20–200 µL pipette; thereafter, the catheter is flushed clear with air (0.2 mL). Used with permission from Norton Thoracic Institute, St. Joseph’s Hospital and Medical Center, Phoenix, AZ

Experimental mice (i.e., groups 1–3) underwent intratracheal instillation of the corresponding substance according to the assigned group once a week for 1 week (n = 3/per group), 3 weeks (n = 3/per group), or 6 weeks (n = 3/per group). A weight-based dose (1 mL/kg) was used at each aspiration for all groups. Each aspiration was performed under sedation with isoflurane. Medication administration for pain control was considered only if acute distress signs were observed. Figure 2 summarizes the aspiration procedure.

#### HCl preparation

The HCL solution was made fresh before each procedure as described elsewhere [6]. In summary, 281 μL of 1N HCl was mixed with 4.72 mL of normal saline to prepare 5 mL of 56.2 mM HCl; the solution was then titrated using 1N HCl (~ 0–50 µL) to obtain a pH of 1.25. The pH was verified using a benchtop electronic pH-mV monitor, and a maximum variation of ± 0.05 was accepted.

#### Gastric contents preparation

Gastric fluid samples collected and stored from patients with GERD who underwent endoscopic procedures at our center were queried from the institutional biobank (IRB approval: PHX-18–500-251–73-18). Further, the samples were pooled and vortexed until homogenized. The pH was measured before each aspiration, and it ranged from 1.25 to 2.75. Human gastric contents were used due to ease of collection over murine samples. A previous study has shown that human gastric fluids can be used in a rodent model without specific effects secondary to xenogeneic aspirate [18].

#### Combination of HCl and gastric fluid

A mixture using 0.1 mL of each of the previously described substances (i.e., ratio of 1:1) was prepared. The resulting fluid was vortexed until homogenized; the pH ranged between 1.75 and 2.50.

### Sample collection and weight assessment

#### Blood sampling

Blood samples for SAbs assessment were collected under sedation from the submandibular vein of each mouse (~ 145–150 μL) at days 0 (baseline), 14, 21, 28, and 35. At day 90, complete blood volume was obtained after euthanasia for SAbs and SAgs measurement. Serum was obtained after whole blood clotting for two hours and centrifugation at 1000 × g for 20 min; thereafter, the samples were frozen at -20 °C until laboratory analysis.

#### Weight assessment

Animals were monitored weekly for weight loss using a small animal digital scale and monitored for distress signs throughout the study.

#### Euthanasia

At day 90, all mice were euthanized following the Guide for the Care and Use of Laboratory Animals. Both lungs were harvested; one was cryopreserved for future research, and the other was fixed in neutralized 10% formalin and sent to the pathology laboratory for histopathological analysis (detailed below).

### Assessment of serum concentration of anti-Col-V and anti-Kα1T

As previously described by our group [16], ELISA plates were coated overnight at 4 °C with either recombinant Kα1T (1 μg/mL) or Col-V (1 μg/mL; Sigma-Aldrich, St. Louis, Missouri) in phosphate-buffered saline and blocked for 2 h with 1% bovine serum albumin. Serum samples from mice were diluted 1:50 for detection of anti-Col-V and anti-Kα1T, then incubated overnight at 4 °C. SAbs were detected using horseradish peroxidase-conjugated goat anti-mouse IgG (Jackson ImmunoResearch Laboratories, West Grove, Pennsylvania). Color was developed using tetramethylbenzidine substrate and read at 450 nm. Antibody concentrations were calculated using standard curves of known concentrations of anti-Kα1T (Santa Cruz Biotechnology, Dallas, Texas) or anti-Col-V (Abcam, Cambridge, United Kingdom); all analyses were performed in triplicate.

### Assessment of Col-V, Kα1T, and NFκB contained in circulating small EVs

#### Small EV isolation

Small circulating EVs (i.e., exosomes) were isolated using a commercial total exosome isolation kit (Invitrogen by Thermo Fisher Scientific, Waltham, MA). After centrifuging serum samples at 2000 × g for 30 min to remove cells and debris, the supernatant was transferred to a new tube for isolation. Isolation reagent was added to the serum samples (0.2 volumes), vortexed, and incubated at 4 °C for 30 min. After incubation, the samples were centrifuged at 10,000 × g for 10 min at room temperature. EVs contained in the pellet were resuspended in 1 × PBS and then filtered through an Ultrafree-MC Filter (0.22 µm pore size; Millipore Sigma, Burlington, Massachusetts) to yield EVs < 220 nm in diameter.

#### Western blotting

Protein contents and levels in small EVs were estimated using a BCA protein estimation kit (Thermo Fisher Scientific). Proteins from EVs (15 µg) were separated under reducing conditions using 4% to 12% gradient Bis–Tris gel (Invitrogen by Thermo Fisher Scientific) electrophoresis and transferred to a polyvinylidene fluoride membrane (Invitrogen by Thermo Fisher Scientific). Further, membranes were probed with antibodies to lung SAgs Col-V (Abcam) and Kα1T (Santa Cruz Biotechnology Inc.) as well as to antibodies against NF-kB (Cell Signaling Technology Inc, Danvers, Massachusetts) as an inflammatory marker, and CD63 (Santa Cruz Biotechnology Inc.) as an EV-specific marker. A nonspecific light-chain immunoglobulin was used as the loading control and detected using peroxidase AffiniPure Goat Anti-mouse IgG light-chain specific antibody (1:10,000) (Jackson ImmunoResearch Laboratories). The blots were developed using an enhanced chemiluminescent immunoblot detection kit (Thermo Fisher Scientific) and read with ImageJ Software (National Institutes of Health) to determine the mean optical density (m.o.d.) of the signal band.

### Histopathological assessment

The lung specimen was embedded in paraffin blocks and cut into 3-mm thick sections for hematoxylin–eosin and trichrome staining. Additional sections were used for immunohistochemistry and were stained with anti-CD3 and anti-CD20 (Thermo Fisher Scientific). A blinded pathologist (M.H.) assessed the most representative set of slides from each subgroup. A semi-quantitative scale was used to grade the loss of alveolar spaces and the alveolar thickness (- none; + mild; +  + moderate; +  +  + severe). Parenchymal fibrosis was graded using a scale from 0 to 100 based on trichrome staining and the modified Ashcroft scale [19]. Morphometric analysis was used to quantify cellular infiltration of CD3 + T cells and CD20 + B cells using high-powered microscopy (i.e., 100 ×). All images were obtained on a Leica microscope (Leica Microsystems, Wetzlar, Germany) at 10 × and 40 × .

### Endpoints

The primary endpoints were the measurement of concentrations of serum SAbs (i.e., anti-Col-V and anti-Kα1T) at day 0 (baseline) and days 14, 21, 28, 35, and 90 after initiation of repetitive aspirations and the quantification of SAg levels (i.e., Col-V and Kα1T contained in circulating EVs) at day 90. *Secondary endpoints* included changes in weight loss as a surrogate of the clinical impact of aspiration, histopathological assessment of lung damage, and measurement of circulating NF-kB contained in small EVs as a marker of inflammation.

### Sample size and power analysis

Using two-sided tests with α error probability of 0.05 and based on a pilot study demonstrating that 50% of patients with GERD presented with abnormal levels of Col-V and Kα1T contained in circulating small EVs [20], we determined that the N needed to achieve induction of SAgs with aspiration of gastric contents in at least 50% of the animals was 21 with a power of 0.80 or 30 with a power > 0.95. Hence, assuming that SAgs and SAbs will be induced with at least one aspiration event, we proposed an experimental design with 27 mice in the intervention arm (3 per subgroup) and 9 controls to achieve a reasonable power > 0.90.

### Statistical Analyses

Continuous variables are reported using means and standard deviations, and categorical variables are reported as counts and proportions, unless otherwise specified. All continuous data, such as antibody concentrations, optical density of the immunoblots, and mice weights were tested for normality using the Shapiro–Wilk test and Q-Q plots. The Kruskal–Wallis or Mann–Whitney U tests were employed as appropriate to assess differences between subgroups using data from at least 3 mice in each experimental subgroup. The Wilcoxon signed-rank test was used to determine differences in SAb concentrations within the same group (i.e., paired samples) at different prespecified time points. Furthermore, the correlations between the concentration of SAbs or SAgs and other covariates were explored using the Spearman's rank correlation coefficient. A significance level (i.e., two-tailed p-value) of 0.05 was set. The statistical analyses were performed blind using SPSS Statistics v29.0 (IBM Corp. Armonk, New York) and GraphPad Prism version 10.2.1 (GraphPad Software, La Jolla, California).

## Results

### Clinical response to aspiration

**Fig. 3:**
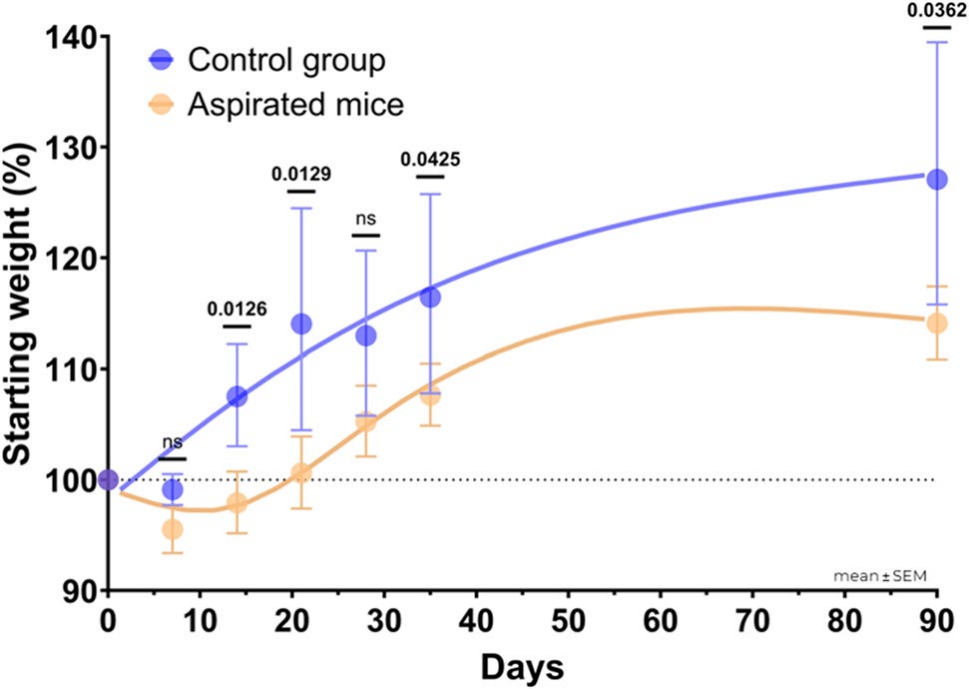
Monitored weight change among aspirated mice (n = 27) and control group (n = 9)

All mice survived the aspiration regimen; no significant distress signs were detected throughout the study period, and euthanasia was not required until the prespecified time point. However, the clinical impact of aspiration was evidenced by changes in animal weight. In the short term, aspirated mice steadily lost weight after the first aspiration, with an average peak weight loss of ∼5% on day 7, and regained their starting weight by day 21. In the long term, aspirated mice had slower weight gain than the control group. Non-aspirated mice had rapid weight gain throughout the experiment as expected for young animals. At day 90, aspirated mice had significantly less weight gain than the control group (14.4 ± 8.3% vs. 27.6 ± 13%, p < 0.05). Figure 3 presents the mean animal weight of both groups. Similarly, when analyzed by subgroups (i.e., type and frequency of aspiration), all of the aspirated subgroups had less weight gain than the control group; however, a trend toward worse tolerance to aspiration was noted among mice aspirated with human gastric contents (Supplementary Material S2).

### SAbs against lung SAgs (anti-Col-V and anti-Kα1T)

**Fig. 4:**
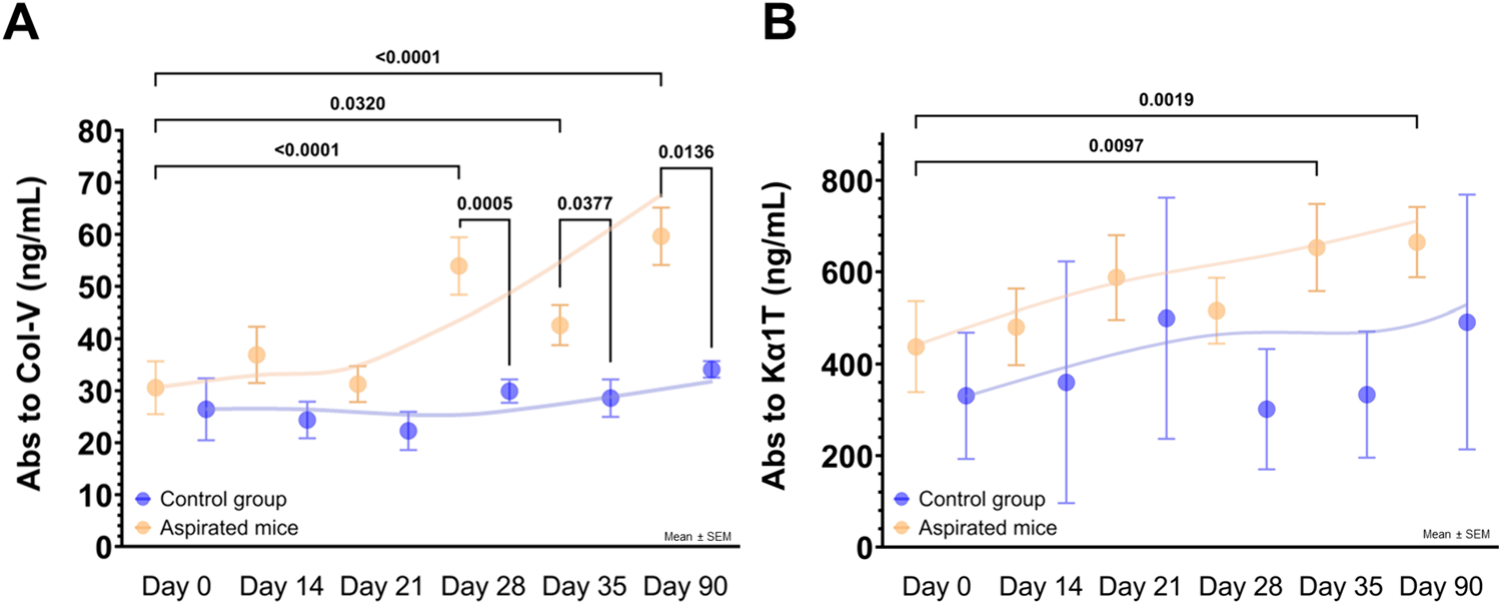
Concentrations over time of antibodies against SAgs (i.e., Col-V and Kα1T) among aspirated mice and controls. **A** Concentrations of anti-Col-V were significantly higher in aspirated mice (n = 27) at 28, 35, and 90 days after the first aspiration compared to the control group (n = 9). Similarly, concentrations among aspirated mice on day 90 were significantly higher than those of baseline concentrations. **B** Anti-Kα1T concentrations did not differ between aspirated mice (n = 27) and controls (n = 9) at any time point; however, compared to the baseline, there was a significant increase of anti- Kα1T concentrations among aspirated mice. Abbreviations: Abs, antibodies; Col-V, collagen type V; Kα1T, K-alpha-1 tubulin; ns, not significant

First, we assessed and compared the induction of autoimmune responses to lung SAgs. Regardless of the frequency or type of substance, mice that were aspirated presented with higher concentrations of anti-Col-V at day 28 (53.9 ± 28.7 ng/mL vs. 29.9 ± 4.5 ng/mL, p < 0.01), day 35 (42.6 ± 19.8 ng/mL vs. 28.6 ± 7.2 ng/mL, p = 0.038), and day 90 (59.7 ± 27.7 ng/mL vs. 34.1 ± 3.2 ng/mL, p = 0.014) than the control group. Moreover, significant differences compared to the basal concentrations of anti-Col-V were noted among aspirated mice at each of the prespecified time points (day 28, p < 0.01; day 35, p < 0.05; day 90, p < 0.01), thus suggesting a window of ∼3–4 weeks to the peak of the primary antibody response (Fig. 4A).

On the other hand, although the mean anti-Kα1T concentration at days 28, 35, and 90 from aspirated mice was higher than that of the control group, these differences did not reach statistical significance. However, compared to the baseline, the anti-Kα1T concentration was higher at day 35 (437.5 ± 473.4 ng/mL vs. 635.3 ± 493.6 ng/mL, p < 0.01) and day 90 (437.5 ± 473.4 ng/mL vs. 665.1 ± 391.2 ng/mL, p < 0.01) in the aspirated mice (Fig. 4B).

When analyzing specific subgroups, as expected, there was no immune response to Col-V in the control group (anti-Col-V baseline concentrations: 26.5 ± 11.9 ng/mL vs. day 90: 34.1 ± 3.2 ng/mL, p = 0.250). However, in most of the mice aspirated with acidic contents, the baseline concentration of anti-Col-V doubled by day 90 and was significantly higher (Supplementary Material S3A). The immune response to Kα1T among the control group was also minimal to zero (anti-Kα1T concentration, baseline: 330.6 ± 275.5 ng/mL vs. day 90: 491.1 ± 555.4 ng/mL, p = 0.250); but among aspirated mice, the response was very heterogeneous (Supplementary Material S3B).

### Circulating small EVs containing lung SAgs (Col-V and Kα1T)

**Fig. 5:**
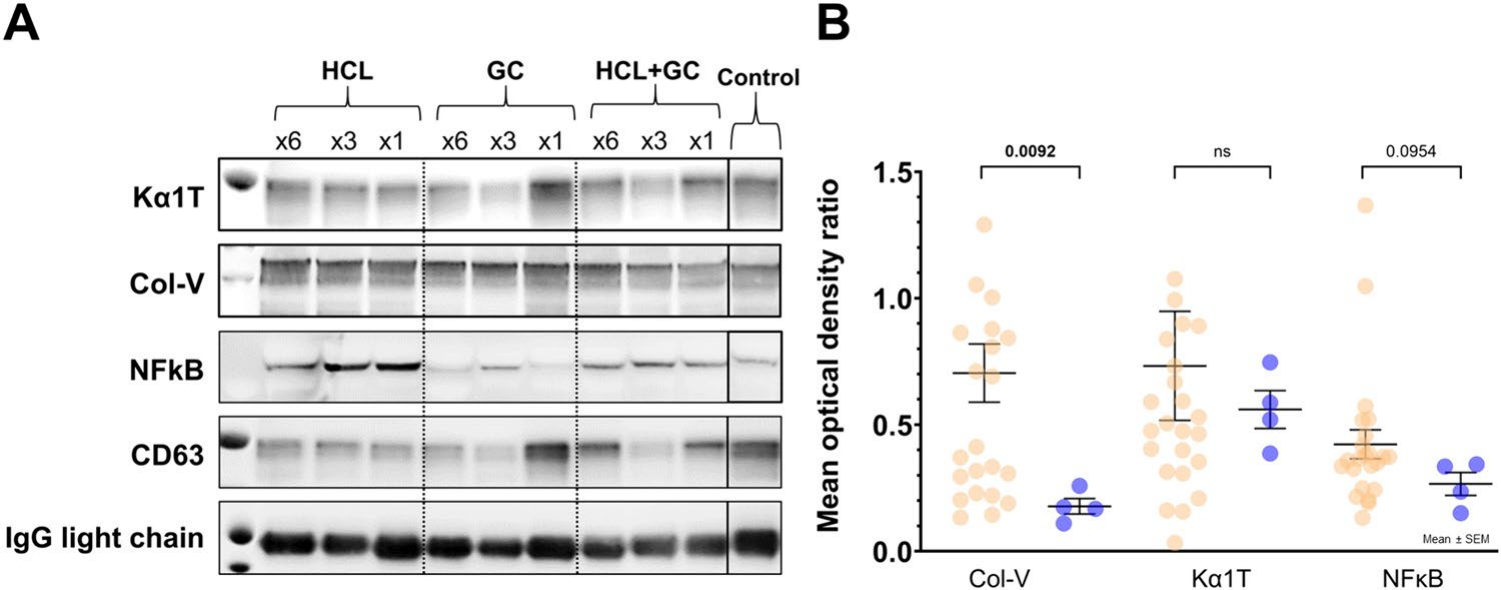
**A** Representative western blots for lung SAgs (i.e., Col-V and Kα1T) and NF-κβ. **B** Densitometry of western blots shows higher levels of small EVs with Col-V among aspirated mice on day 90 (n = 27) compared to the control group (n = 4; five samples were insufficient to perform small EVs isolation). Abbreviations: Col-V, collagen type V; EVs, extracellular vesicles; GC, gastric contents; HCL, hydrochloric acid; IgG, immunoglobulin G; Kα1T, K-alpha-1 tubulin; NF-κβ, nuclear factor κB; ns, not significant

Next, we isolated small EVs from the serum samples of each animal. The size of the EVs was limited to < 220 nm by filtering methods. The resulting EVs were analyzed by western blot (Fig. 5A) using antibodies to lung SAgs (i.e., Kα1T and Col-V) as well as to a nonspecific inflammatory transcription factor (i.e., NF-κB). In aspirated mice, circulating small EVs contained significantly higher levels of Col-V at day 90 than those from the control group (0.7 ± 0.56 vs. 0.18 ± 0.6 mean optical density [m.o.d.], p = 0.009); moreover, a trend toward increased levels of NF-κB within EVs was noted (0.42 ± 0.27 vs. 0.27 ± 0.09 m.o.d., p = 0.095). In contrast, aspirated mice and control mice had similar levels of Kα1T (0.73 ± 1.1 vs. 0.56 ± 0.15 m.o.d., p = 0.825; Fig. 5B).

**Fig. 6:**
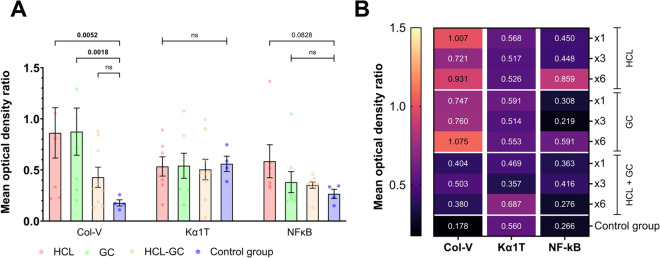
Assessment of Col-V, Kα1T, and NF-κβ contained in circulating small extracellular vesicles at day 90. **A** The mean optical density ratio for Col-V among mice aspirated with HCL or GC was significantly higher than that of the control group. **B** Differences between subgroups are highlighted in the heatmap. Mice with aspiration of HCL or GC, regardless of the number of aspiration events, presented with higher levels of Col-V; moreover, relatively higher levels of NF-κB were noted in groups that underwent more aspirations using either HCL or GC. Abbreviations: Col-V, collagen type V; GC, gastric contents; HCL, hydrochloric acid; Kα1T, K-alpha-1 tubulin; NF-κB, nuclear factor κB; ns, not significant

Furthermore, the densitometry of western blots demonstrated some differences according to the type of aspirated substance. The highest levels of Col-V compared to controls were found among mice aspirated with HCl (0.86 ± 0.65 vs. 0.18 ± 0.6 m.o.d, p < 0.05) and human gastric contents (0.87 ± 0.65 vs. 0.18 ± 0.6 m.o.d, p < 0.05). Whereas the levels of NF-κB contained in EVs were discretely higher than controls only in mice aspirated with HCl (0.58 ± 0.39 vs. 0.27 ± 0.09 m.o.d, p = 0.083). No differences in Kα1T levels were observed between subgroups (Fig. 6A and 6B).

### Histopathological assessment of lung damage

**Fig. 7:**
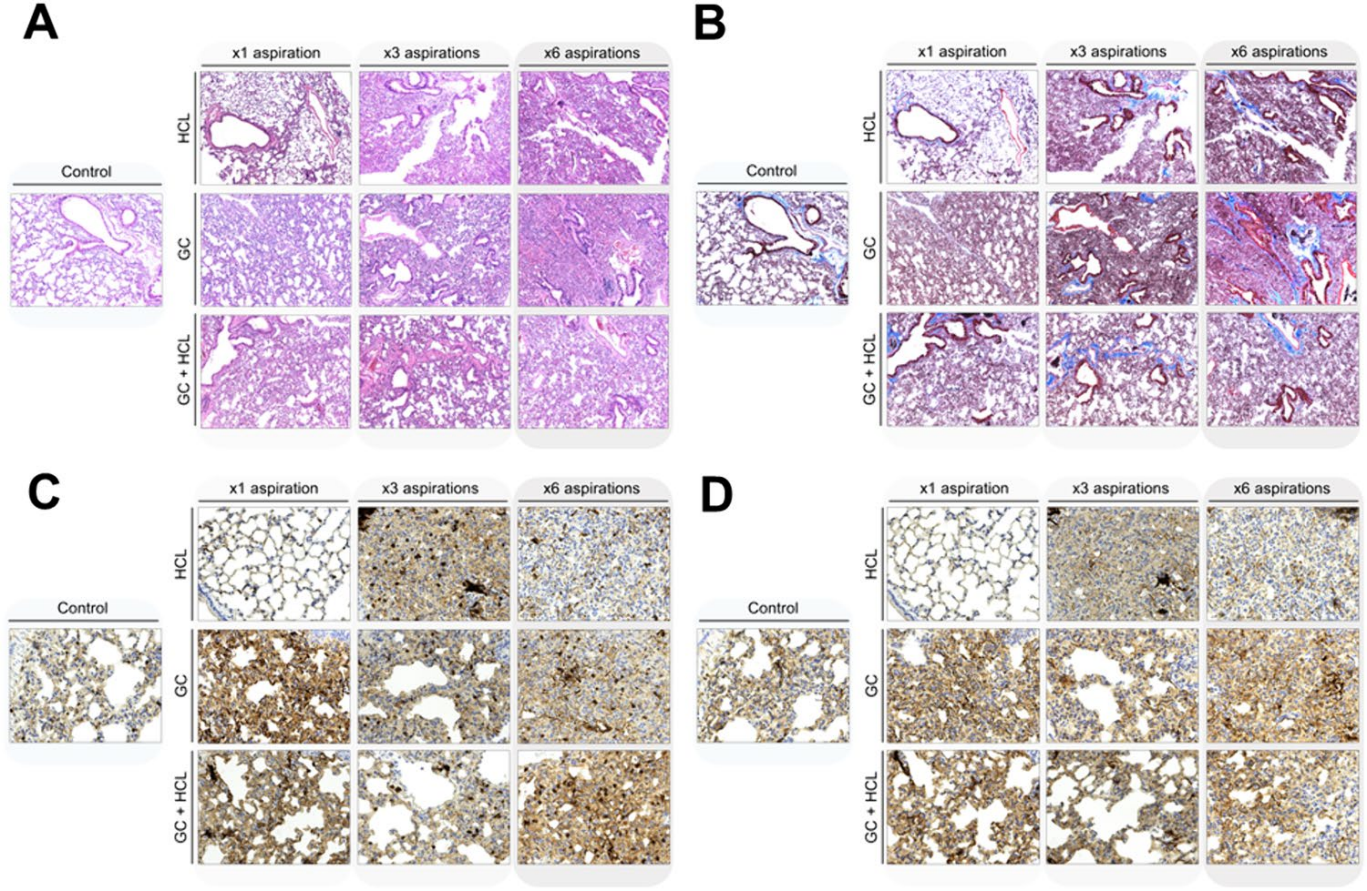
Histopathological assessment of lung sections at 90 days after first aspiration. **A** Hematoxylin and eosin staining, 10 × . **B** Trichrome staining, 10 × . **C** Immunostaining to CD3, 40 × . **D** Immunostaining to CD20, 40 ×

Lung sections stained with hematoxylin and eosin from each subgroup are presented in Fig. 7A. All lungs from aspirated mice presented evidence of pulmonary injury as indicated primarily by structural collapse (i.e., loss of alveolar spaces) or increased alveolar thickness. Moreover, regardless of the type of aspirated substance, the severity of injury increased as the animals underwent more aspiration events. In contrast, a preserved parenchymal architecture was noted in all of the controls. When analyzing representative lung sections stained with trichrome, there was evidence of early parenchymal fibrotic changes in some aspirated mice (Fig. 7B), especially those that underwent 3 or 6 aspirations (the extent of fibrosis ranged from 10–30%). The modified Ashcroft scale score was zero in all control mice, whereas it ranged from 0 to 2 points among aspirated mice. Table 1 summarizes the histopathological findings of each subgroup.

**Table 1:** Summary of histopathological findings by number of aspirations and aspirated substance

Study group	Loss of alveolar spaces	Parenchymal fibrosis	Alveolar thickness	Modified ashcroft scale	Highest CD3 + count	Highest CD20 + count
Control	–	0%	–	0	32/hpf	1/hpf
HCL × 1	+	0%	–	0	55/hpf	1/hpf
HCL × 3	+ +	20%	+	2	63/hpf	5/hpf
HCL × 6	+ + +	10%	+	2	49/hpf	2/hpf
GC × 1	+	10%	–	1	49/hpf	2/hpf
GC × 3	+ +	20%	–	2	44/hpf	2/hpf
GC × 6	+ + +	30%	+	2	45/hpf	1/hpf
HCL + GC × 1	+	0%	–	0	32/hpf	1/hpf
HCL + GC × 3	+	10%	+	2	55/hpf	1/hpf
HCL + GC × 6	+ +	10%	–	1	55/hpf	1/hpf

Abbreviations: - none,  +  mild,  +  +  moderate,  +  +  +  severe, GC, gastric contents, HCL, hydrochloric acid, hpf, high power field

Immunohistochemical staining of representative lung sections was performed with antibodies to CD3 and CD20, followed by morphometric analysis and estimation of cellular infiltration. The maximum count of CD3 + T cells was 32 per high-powered field among controls, and none of the aspirated subgroups presented with a lower maximum count. Importantly, the CD3 + T cell infiltration was diffuse in the alveolar spaces and parenchyma (Fig. 7C). Moreover, the reactivity to CD20 was low, and the count of B cells was relatively similar across all the groups (i.e., maximum count per high-powered field ranged between 1 and 5 cells) (Fig. 7D).

Further, through exploratory analysis, we identified positive, strong, and significant correlations between the levels of NF-κB contained in isolated EVs and alveolar thickness (rs = 0.866, p < 0.05), between the amount of Col-V and loss of alveolar spaces (rs = 0.784, p < 0.05) and alveolar thickness (rs = 0.693, p < 0.05), and between the concentration of anti-Col-V at day 90 and the count of CD3 + T cells (rs = 0.795, p < 0.05). Table 2 summarizes the correlations between immune responses and lung damage that reached a p-value < 0.1.

**Table 2:** Correlations between markers of immune response and histopathological findings showing lung damage

Correlation	Spearman's rho (95% CI)	p-value
NF-κB contained in EVs and alveolar thickness	0.8660 (0.4567, 0.9727)	**0.0025**
Antibodies to Col-V and CD3 + count	0.7950 (0.2553, 0.9569)	**0.0104**
Col-V contained in EVs and loss of alveolar spaces	0.7840 (0.2277, 0.9544)	**0.0124**
Col-V contained in EVs and alveolar thickness	0.6928 (0.0295, 0.9324)	**0.0386**
NF-κB contained in EVs and modified Ashcroft grade	0.6522 (−0.0446, 0.9221)	0.0570
Col-V contained in EVs and modified Ashcroft grade	0.6336 (−0.0763, 0.9172)	0.0670

Only correlations with p-value < 0.1 are presented. Bold p-values are statistically significant (p < 0.05). Abbreviations: CI, confidence interval; Col-V, collagen type V; EVs, extracellular vesicles; NF-κB, nuclear factor κB

## Discussion

This exploratory study elucidates the complex relationship between GERD, aspiration, and lung damage. With the use of a murine model of repetitive aspiration events simulating GERD-induced lung injury, we demonstrated that aspiration of acidic contents leads to an early immune response and that increased levels of specific serum markers (i.e., antibodies against Col-V as well as Col-V contained in circulating small EVs) correlates with objective evidence of lung damage. Hence, this opens an exciting avenue for *i)* understanding the immune response secondary to aspiration and its role in the development of chronic lung diseases and *ii)* exploring the use of these serum markers as a noninvasive method for early identification of patients with GERD who may have subclinical lung damage.

Historically, GERD has been recognized as a plausible cause of chronic respiratory disorders such as chronic cough, asthma, bronchitis, and idiopathic fibrosis, among others [2, 21]; however, the current pathophysiological mechanisms to explain respiratory manifestations of GERD have been limited to two theories. The first one is known as the “reflux theory” and the second as the “reflex theory” [22]. Whereas the first one proposes that refluxed gastric contents cause direct mechanical injury of the respiratory epithelium (due to low pH or the presence of peptidases such as pepsin), the second one proposes that the respiratory symptoms result from a vagal-mediated esophageal–tracheobronchial reflex facilitated by hyper-responsiveness to the refluxate [22, 23]. Notably, our study was conceptualized based on studies of aspiration-mediated allograft rejection after lung transplantation [12, 16], and our findings suggest that extrapolation to non-lung transplant patients is feasible; hence, we propose a plausible third pathophysiological mechanism based on specific immune responses elicited by aspiration to explain respiratory manifestations of GERD. This mechanism, in which parenchymal damage with direct lung injury is compounded by an immune-mediated response, is supplementary to the reflux theory.

We consider that a patient-specific respiratory condition cannot be reduced to a single unique mechanism; instead, the interaction of multiple pathogenic pathways (i.e., whether mechanical, neurological, or immunological) may be necessary, thus resulting in the known broad spectrum of respiratory disease phenotypes and clinical manifestations among patients with GERD. For example, a predominant vagal-mediated reflex hyper-responsiveness may better explain the clinical manifestations of a patient with GERD and asthma (i.e., cough or bronchospasm). In contrast, the usually late symptomatic presentation of respiratory symptoms in a patient with idiopathic pulmonary fibrosis may be explained by a predominant mechanical injury of the epithelium and superimposed immune response.

Furthermore, our results indicate that circulating small EVs with lung SAgs (i.e., Col-V) and their corresponding antibodies (i.e., anti-Col-V) might have a role as markers of lung injury. Induction of anti-Col-V occurred in most of the aspirated subgroups regardless of the frequency or type of substance, which indicates that even a single aspiration event of acidic contents may lead to an autoimmune response in some individuals.

The time needed to detect an antibody response was 3 to 4 weeks, somewhat longer compared to murine models of lung transplantation evaluating the pattern of de novo development of antibodies to lung SAgs [16, 24]. The severity of the injury may explain this prolongation of antibody induction; in the case of repetitive small-dose aspiration (i.e., as is believed to occur in patients with GERD), the exposure to SAgs occurs progressively and in a controlled manner, whereas in the lung transplant setting, any insult to allograft epithelium may be considered significant, resulting in increased reactivity.

The reasons why aspirated mice and controls had similar levels of Kα1T in circulating small EVs and comparable serum anti-Kα1T concentrations are unclear. However, the nature of the SAgs may provide some explanations. Immune responses to Kα1T may be more heterogeneous than those against Col-V; although anti-Kα1T has been associated with lung fibrosis and allograft dysfunction after transplantation [12, 25–27], it has also been linked to other diseases such as small cell lung carcinoma, breast cancer [28], postcardiac transplant cardiomyopathy [29], and relapsing polychondritis [30]. On the other hand, the exposure of normally sequestered Col-V has been consistently associated with pulmonary fibrosis (e.g., up to 60% of lung transplant patients with pulmonary fibrosis present with T cells reactive to anti-Col-V, and roughly 50% present with abnormal expression of anti-Col-V) [25, 27, 31]. Therefore, the distinctive immunogenic properties of Col-V may contribute to a more pronounced, specific, and sustained immune response to aspiration of acidic contents than those of Kα1T.

The frequency of aspiration and the immune response might determine the extent of lung damage. We found that structural changes including loss of alveolar spaces, increased alveolar thickness, and early evidence of fibrosis occurred more frequently in mice that underwent more aspiration events regardless of the substance used. These findings align with those from other animal models evaluating lung injury after aspiration of acidic contents [10]. Moreover, we identified a trend towards higher CD3 + T cell counts among aspirated mice, which are a potential source of IL-17 (i.e., Th17 cells and monocyte/macrophage accessory cells), a proinflammatory cytokine that has been associated with pulmonary fibrosis and progressive airway obliteration [32, 33].

We believe that studying the described immune mechanism in humans helps to elucidate the underlying pathophysiology of GERD-mediated lung injury. These humoral markers have been detected, measured, and associated with worse outcomes in lung transplant recipients with GERD [34]. Our ongoing research efforts are focused on the role of these markers in non-lung transplant patients with GERD with or without overt respiratory symptoms [35]. Although it is premature to predict, these findings could yield important clues as to how lung damage occurs after aspiration and, more importantly, identify measurable markers for lung damage in patients with a long-standing history of GERD.

Our study has some limitations, including a relatively small sample size, which limited the interpretation of results and precluded further statistical analysis. Notably, there was a learning curve for the performance of aspirations during the development of the animal model, which could result in inadequate aspiration during the initial procedures; however, the clinical impact of each procedure was evaluated by weighing the animals, and when comparing subgroups, there was a difference between aspirated mice and controls from the first week. There was also a lack of histological assessment and measurement of SAgs contained in circulating EVs until the final time point at day 90, which limited the evaluation of morphological and immune responses of lung sections after acute aspiration as well as the kinetics of SAgs after aspiration. Despite demonstrating that either a single episode or multiple episodes of aspiration could initiate an autoimmune cascade and induce early evidence of fibrosis, we could not confirm the time required for significant remodeling of lung parenchyma (i.e., mature fibrosis); hence, future studies will consider longer interventions and follow-ups. Furthermore, despite using the same mice strain, the immune response and reactivity to the same stimuli may differ between each biological system. Finally, the pulmonary function of mice was not evaluated, and all of the correlations between immune markers and lung damage were based on pathological findings; however, in the clinical setting, it would be more relevant to understand the relationship between patient symptoms and the immune response to lung aspiration.

## Conclusion

In summary, we have developed a murine model that allows for the study of repetitive aspiration events leading to GERD-induced lung injury, which simulates to a certain degree the human condition (i.e., nonselective, silent, and repetitive micro-aspiration rather than unique and selective large volume reflux aspiration). Our results suggest that aspiration of acidic contents leads to an early immune response (i.e., increased levels of Col-V contained in circulating small EVs and the development of antibodies against Col-V) and a relatively proinflammatory state (i.e., determined by a slight increase of NF-κB contained in EVs), which may provide another pathophysiological explanation of how GERD contributes to chronic respiratory conditions. These findings may be relevant for developing targeted therapies or patient-tailored management strategies to mitigate, or even reverse, existing damage to prevent the progression of chronic respiratory complications in patients with GERD. Moreover, these humoral markers may serve as noninvasive biomarkers for the early detection of asymptomatic lung damage in patients with GERD, allowing prompt and adequate treatment (e.g., mechanical control over medical therapy). Translation to the clinical setting is highly desirable to confirm these findings.

## Supplementary Information

Below is the link to the electronic supplementary material.

Supplementary file1 (DOCX 889 KB)

## Data Availability

All records related to animal work and data collected during the study are stored in either a physical or a digital file. Please contact the corresponding author to access any data; an approval from the local IACUC may be necessary.

## Declarations

### Meeting Presentation

The abstract of this manuscript was presented as a podium presentation at the 2024 annual meeting of the Society of American Gastrointestinal and Endoscopic (SAGES), April 17th-20th, 2024. Cleveland, OH, USA.
