# Supplementary material for: Noninvasive biomarkers for the detection of GERD-induced pulmonary injury
Source: Surg Endosc. 2024 Sep 12;38(12):7491–502. doi: 10.1007/s00464-024-11180-4 (PMC11615114; doi:10.1007/s00464-024-11180-4)

**SUPPLEMENTARY MATERIAL**

**Supplementary material S1.** The ARRIVE Essential 10 checklist (from the ARRIVE 2.0 guidelines).

| **ARRIVE Essential 10** | | | |
| --- | --- | --- | --- |
| Study design | 1 | For each experiment, provide brief details of study design including: a. The groups being compared, including control groups. If no control group has been used, the rationale should be stated. b. The experimental unit (e.g., a single animal, litter, or cage of animals). | Check – a and b are reported in “Mice and study groups” section. |
| Sample size | 2 | a. Specify the exact number of experimental units allocated to each group, and the total number in each experiment. Also, indicate the total number of animals used. b. Explain how the sample size was decided. Provide details of any a priori sample size calculation, if done. | Check – a is reported in “Mice and study groups” section and b in “Sample size and power calculation” section. |
| Inclusion and exclusion criteria | 3 | a. Describe any criteria used for including and excluding animals (or experimental units) during the experiment, and data points during the analysis. Specify if these criteria were established a priori. If no criteria were set, state this explicitly. b. For each experimental group, report any animals, experimental units, or data points not included in the analysis and explain why. If there were no exclusions, state so. c. For each analysis, report the exact value of *n* in each experimental group. | Check – a is reported in “Methods” section, b in “Sample size and power calculation” section, and c across the manuscript or figures for each experiment. |
| Randomization | 4 | a. State whether randomization was used to allocate experimental units to control and treatment groups. If done, provide the method used to generate the randomization sequence. b. Describe the strategy used to minimize potential confounders such as the order of treatments and measurements, or animal/cage location. If confounders were not controlled, state this explicitly. | Check – a N/A, and b is described in “Methods” section. |
| Blinding | 5 | Describe who was aware of the group allocation at the different stages of the experiment (during the allocation, the conduct of the experiment, the outcome assessment, and the data analysis). | Check – allocation group was know by the investigators during the experiment however, the statistical analysis were done in a blinded fashion, and is described in “Statistical Analyses” section. |
| Outcome measures | 6 | a. Clearly define all outcome measures assessed (e.g., cell death, molecular markers, or behavioral changes). b. For hypothesis-testing studies, specify the primary outcome measure, i.e., the outcome measure that was used to determine the sample size. | Check – a and b are reported in “Methods” section. |
| Statistical methods | 7 | a. Provide details of the statistical methods used for each analysis, including software used. b. Describe any methods used to assess whether the data met the assumptions of the statistical approach, and what was done if the assumptions were not met. | Check – a and b are reported in “Statistical Analyses” section. |
| Experimental animals | 8 | a. Provide species-appropriate details of the animals used, including species, strain and substrain, sex, age or developmental stage, and, if relevant, weight. b. Provide further relevant information on the provenance of animals, health/immune status, genetic modification status, genotype, and any previous procedures. | Check – a and b are reported in “Mice and study groups” section. |
| Experimental procedures | 9 | For each experimental group, including controls, describe the procedures in enough detail to allow others to replicate them, including: a. What was done, how it was done, and what was used. b. When and how often. c. Where (including detail of any acclimatization periods). d. Why (provide rationale for procedures). | Check – a, b, c and d are reported in “Methods” section as well as summarized and presented in Figure 1 (study flow diagram). |
| Results | 10 | For each experiment conducted, including independent replications, report: a. Summary/descriptive statistics for each experimental group, with a measure of variability where applicable (e.g., mean and SD, or median and range). b. If applicable, the effect size with a confidence interval. | Check – a and b are reported in “Results” section as well as corresponding figures. |

Explanations and examples for items 1 to 10 are available in the E&E document and on the website at [https://www.arriveguidelines.org](https://www.arriveguidelines.org/). **Abbreviations: ARRIVE**: Animal Research: Reporting of In Vivo Experiments; **E&E:** Explanation and Elaboration.

**Supplementary material S2.** Monitored weight change among aspirated mice and controls (n=9) according to the aspirated substance and frequency (each aspirated subgroup, n=3). A) Representation by aspirated substance. B) Representation by number of aspiration events. Abbreviations: GC, gastric contents; HCL, hydrochloric acid.


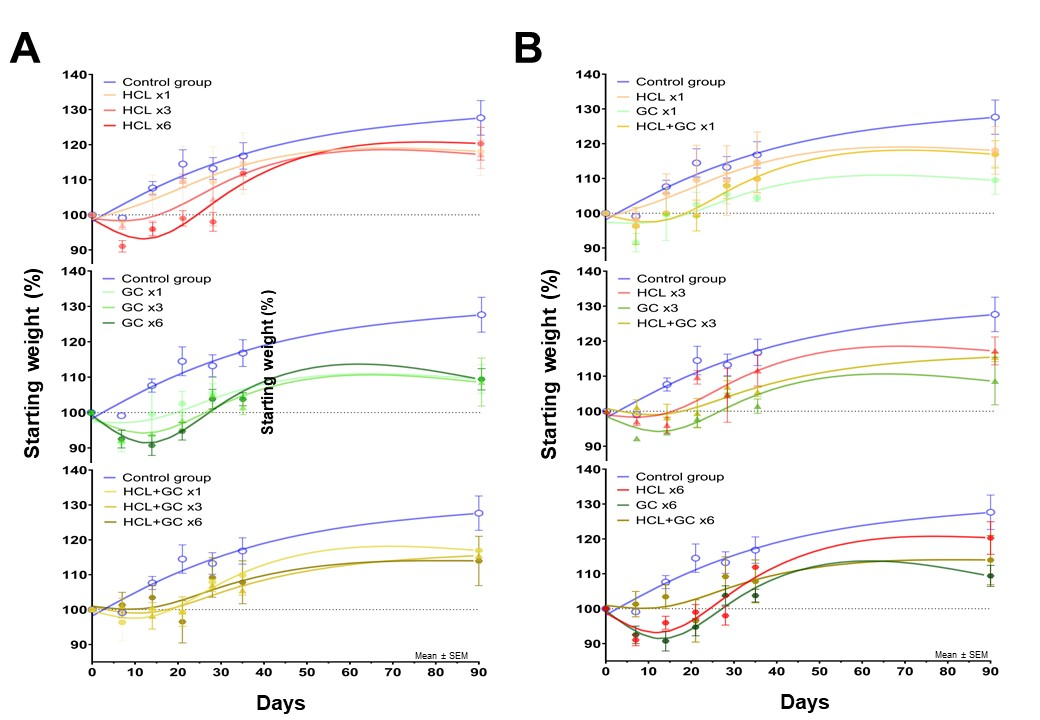


**Supplementary material S3.** Assessment of circulating concentrations of anti-Col-V and anti-Kα1T. The heatmaps highlight the subgroups (n=3) presenting higher mean concentrations of each antibody ([A] anti-Col-V, and [B] anti- Kα1T) and the control group (n=9) at the pre-specified time points according. Abbreviations: Abs, antibodies; Col-V, collagen type V; GC, gastric contents; HCL, hydrochloric acid; Kα1T, K-alpha 1 tubulin.


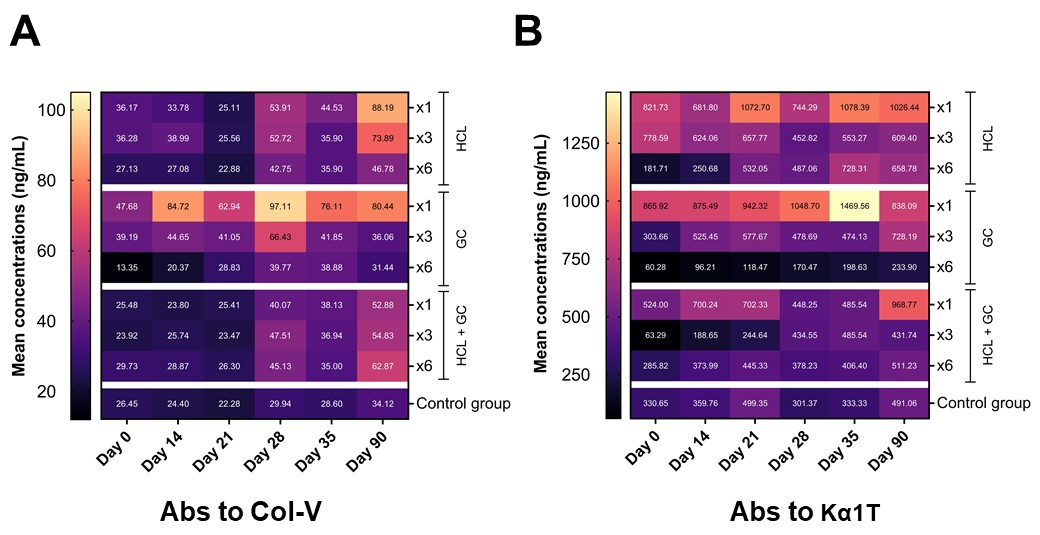

Supplement: Supplementary file 1 — Supplementary file1 (DOCX 889 KB) [file 464_2024_11180_MOESM1_ESM.docx]
